# Local Recurrence Following Total Marrow Radiation: Implications for Clinical Target Delineation

**DOI:** 10.7759/cureus.10592

**Published:** 2020-09-22

**Authors:** Jared H Hara, Kang-Hyun Ahn, Bulent Aydogan, Matthew Koshy

**Affiliations:** 1 Department of Radiation and Cellular Oncology, University of Chicago Medical Center, Chicago, USA; 2 Department of Radiation Oncology, University of Illinois Hospital and Health Sciences System, Chicago, USA

**Keywords:** radiation oncology, hematology-oncology, allogeneic bone marrow transplant, bone marrow failure, myeloid sarcoma, total marrow irradiation, volumetric-modulated arc therapy, chronic myelogenous leukemia

## Abstract

Total marrow radiation is an emerging treatment modality used in patients undergoing stem cell transplantation. We present a rare case of a patient undergoing total marrow irradiation with concurrent ablative stem cell transplantation with local failures in two out-of-field areas that were not included in the clinical target volume A 31-year-old female patient initially presented with abdominal pain secondary to chronic myelogenous leukemia. She underwent dasatinib treatment for years, but subsequently developed recurrence and underwent consolidation systemic chemotherapy followed by allogeneic stem cell transplantation with adjuvant total marrow irradiation. Several months later, she noticed increased left jaw swelling and dysphagia with associated right ankle swelling. Biopsy of the right ankle and left mandible were consistent with recurrent myeloid sarcoma. This case report suggests that inclusion of the mandible and lower extremities may be necessary when performing total marrow radiation.

## Introduction

Recent advances have made the use of beams of varying intensity possible by modulating the radiation delivery according to the optimized plan generated based on a CT image of the patient. These approaches, known as intensity-modulated arc therapy (IMRT) or volumetric-modulated arc therapy (VMAT), allow the high-dose region to be conformed to the shape of the target while reducing the dose to normal organs. IMRT and VMAT represent major advancements in the delivery of therapeutic radiation and are commonly used in the treatment of most solid tumors [1].

VMAT can be used to deliver radiation to the bone marrow while sparing normal organs. Our group developed and clinically implemented a linac-based IMRT and now VMAT total marrow irradiation (IMTMI and VMTMI, respectively) [2-4]. It has been used successfully to deliver radiation as part of the conditioning regimen in autologous transplant for myeloma [5,6] and as part of reduced intensity allogeneic hematopoietic stem cell transplantation (HSCT) for leukemia [7]. We have previously demonstrated the preclinical and clinical safety for total marrow radiation as a conditioning regimen in patients undergoing stem cell transplantation [2,4,8-10]. Our regimen of 9 Gy of TMI with myeloablative FluBu chemotherapy has been previously published in a phase I clinical trial [11] and is currently being tested in a phase II study (NCT03121014) [12].

Myeloid sarcoma

Myeloid sarcoma (also known as granulocytic sarcoma, myeloblastoma, or chloroma) is a rare presentation of myeloid leukemia and characterized by prominent extramedullary disease [13]. It may present simultaneously or precede bone marrow disease and is sometimes seen in relapse. It typically occurs as either cutaneous or gingival infiltration by leukemic cells and is often seen when there is a prominent monocytic component to the leukemia. Sites of isolated myeloid sarcoma include bone, periosteum, soft tissues, and lymph nodes. Less commonly involved sites include the orbit, intestine, mediastinum, epidural region, uterus, and ovary [14,15].

## Case presentation

The patient is a 31-year-old woman who presented in 2011. She was evaluated in the emergency department for abdominal pain and was found to have an elevated white blood cell count. She subsequently underwent a bone marrow biopsy, which revealed chronic myelogenous leukemia (CML). She was placed on imatinib and then dasatanib for two years.

In April 2019, she was noted to have swelling of a gingival mass, and a bone marrow biopsy revealed hypercellular bone marrow >90% with trilineage hematopoiesis and marked myeloid hyperplasia consisting of blasts 2%, eosinophils 3%, and basophils 1%. The specimen was noted to have markedly decreased to absent marrow iron stores and no ringed sideroblasts. Cytogenetics were consistent with 46,XX, del(5)(q13q22), t(9;22)(q34;q12.2)(11)/46, sl, t(3;21)(q26;q22), t(7;7)(p22;q21)(2)/47, sd11,+8(2)/48, sdl2, der(22)t(9;22)(4)/46, XX(1). Molecular testing for FMS-like tyrosine kinase 3 (FLT3), nucleophosmin (NPM1), CD117 tyrosine-protein kinase KIT (c-KIT), and CCAAT/enhancer-binding protein alpha (CEBPA) was negative. No P210 bcrabl+ABLE T315I mutation was identified. Overall, these findings were concerning for extramedullary myeloid sarcoma consistent with myeloid blast phase of CML, bcr-abl+.

She underwent induction chemotherapy with imatinib. Subsequently, a bone marrow biopsy in June 2019 revealed a complete response. She was then recommended for allogeneic stem cell transplantation (fludarabine (30 mg/m^2^), cyclophosphamide (14.5 mg/adjusted bodyweight/day), mesna (14.5 mg/adjusted bodyweight/day)) with TMI of 9 Gy per our institutional phase 2 protocol (NCT03121014) [12]. She tolerated her treatment with no unexpected side effects or toxicities.

Volumetric-modulated total marrow irradiation

A custom whole-body frame was created to immobilize the patient, and a full body CT was obtained. The clinical target volume consisted of all the bones in the body from head to the mid-femur, plus a 3.0-mm margin. However, in order to improve sparing of the oral cavity and reduce the incidence of acute toxicities it had been institutional practice to exclude the mandible from the total marrow clinical target volume. Furthermore, due to treatment field length considerations the mid-humerous and mid-femur were typically excluded from the clinical target volumes. The TMI technique consisted of three plans: the head and neck, the chest, and the pelvis, each with 3-6 VMAT arcs to cover the targets with more than 720° modulation potential. The first plan encompassed the bones in the head and neck including the cranium and cervical vertebral bodies (C1 through C7). The chest plan delivered a treatment to a field starting from the inferior portion of the head and neck plan and extended a few centimeters beyond the ribs to include the sternum, ribs, and thoracic vertebral bodies (T1 through T12). The bones in the pelvic field included os coxae, femoral head, and lumbar vertebral body L1 through L5. Three isocenter plans are optimized and calculated using base plan function in the Eclipse treatment planning system version 15.5 (Varian Medical Systems, Palo Alto, CA) in order to ensure dose homogeneity in junctions. The plan was prescribed to ensure a 95% planning target volume dose coverage with the prescription dose with a maximum dose of 140% in the target.

**Figure 1:**
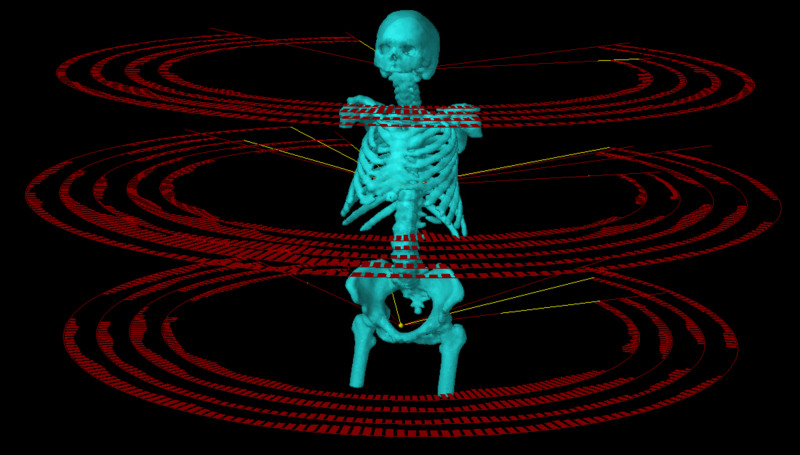
Patient’s planning target volume (PTV) and treatment fields (arcs) of a linac-based volumetric-modulated arc therapy (VMAT) total marrow irradiation plan with three isocenter setups.

**Figure 2:**
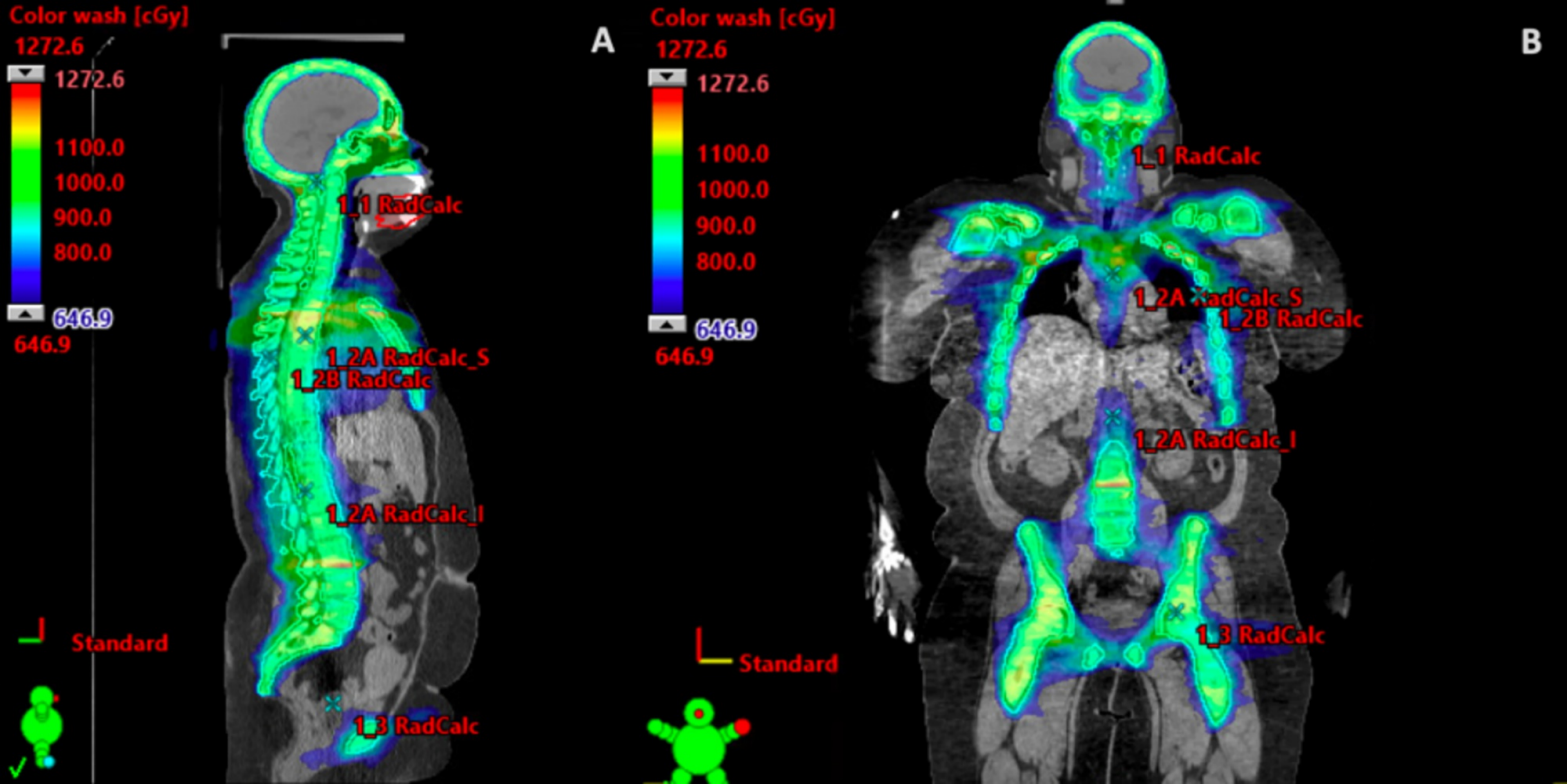
Patient’s total marrow irradiation treatment plan with a (A) sagittal view and (B) coronal view.

The organs at risk included the lungs, heart, liver, kidneys, bowels, brain, eyes, oral cavity, and lenses and were contoured by the radiation oncology physician on axial CT images. Per day, a 3-Gy dose of TMI was administered in two fractions (1.5 Gy twice a day) (Figures 1, 2). Doses of radiation were separated by at least six hours. Radiation was delivered using a linear accelerator.

**Figure 3:**
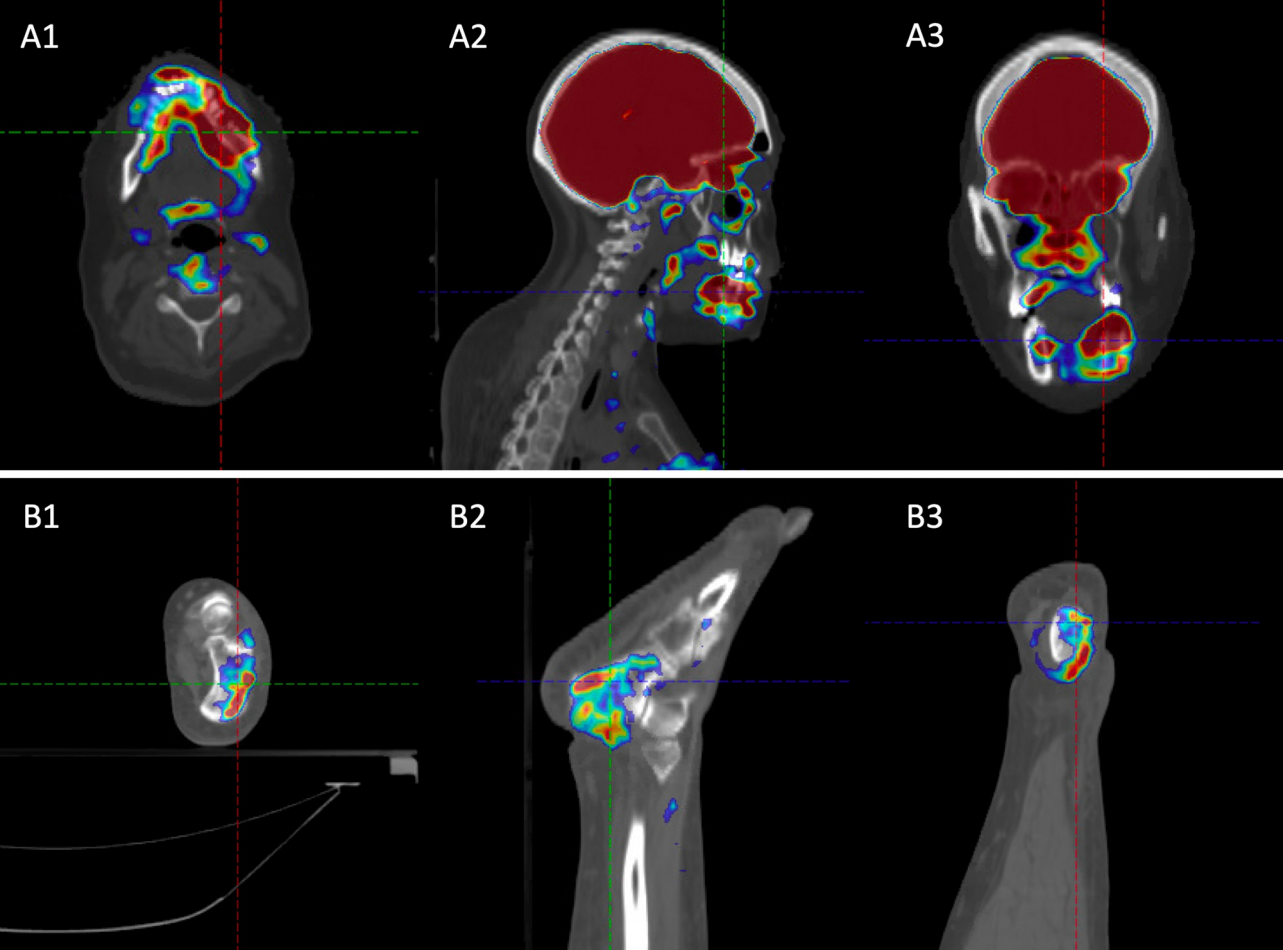
Representative images of positron emission tomography (PET)/CT at relapse, which shows the (A) mandibular and (B) right ankle 18-F fluorodeoxyglucose (FDG) avidity using an (1) axial view, (2) sagittal view, and (3) coronal view.

Approximately six months later, she was found to have a large soft tissue mass surrounding the left mandibular body radiographically measuring 7.0 cm, which was consistent with the location of her prior gingival mass. At the time, she reported pain, tenderness, inability to chew, and left-sided trismus. A left mandibular biopsy revealed CML recurrence consistent with recurrent myeloid sarcoma. She was also noted to have right-sided ankle swelling and associated pain. CT and subsequent positron emission tomography (PET)/CT studies revealed lytic osseous infiltration of the left mandible and right ankle with associated 18-F fluorodeoxyglucose avidity (Figure 3).

She was subsequently treated with cytarabine and mitoxantrone and continued on dasatinib with excellent response. Her left mandibular and right ankle symptoms resolved as well as the associated swelling. She was then treated with consolidative radiation to 25 Gy in 10 fractions to both involved sites, i.e., right ankle and mandible. She tolerated the treatment well overall with grade 2 oral mucositis, which was managed with viscous lidocaine. Following the completion of treatment, her left jaw trismus, edema, and pain as well as her right ankle edema clinically resolved. However, three months following the radiation, she was noted to have relapse of disease.

## Discussion

**Figure 4:**
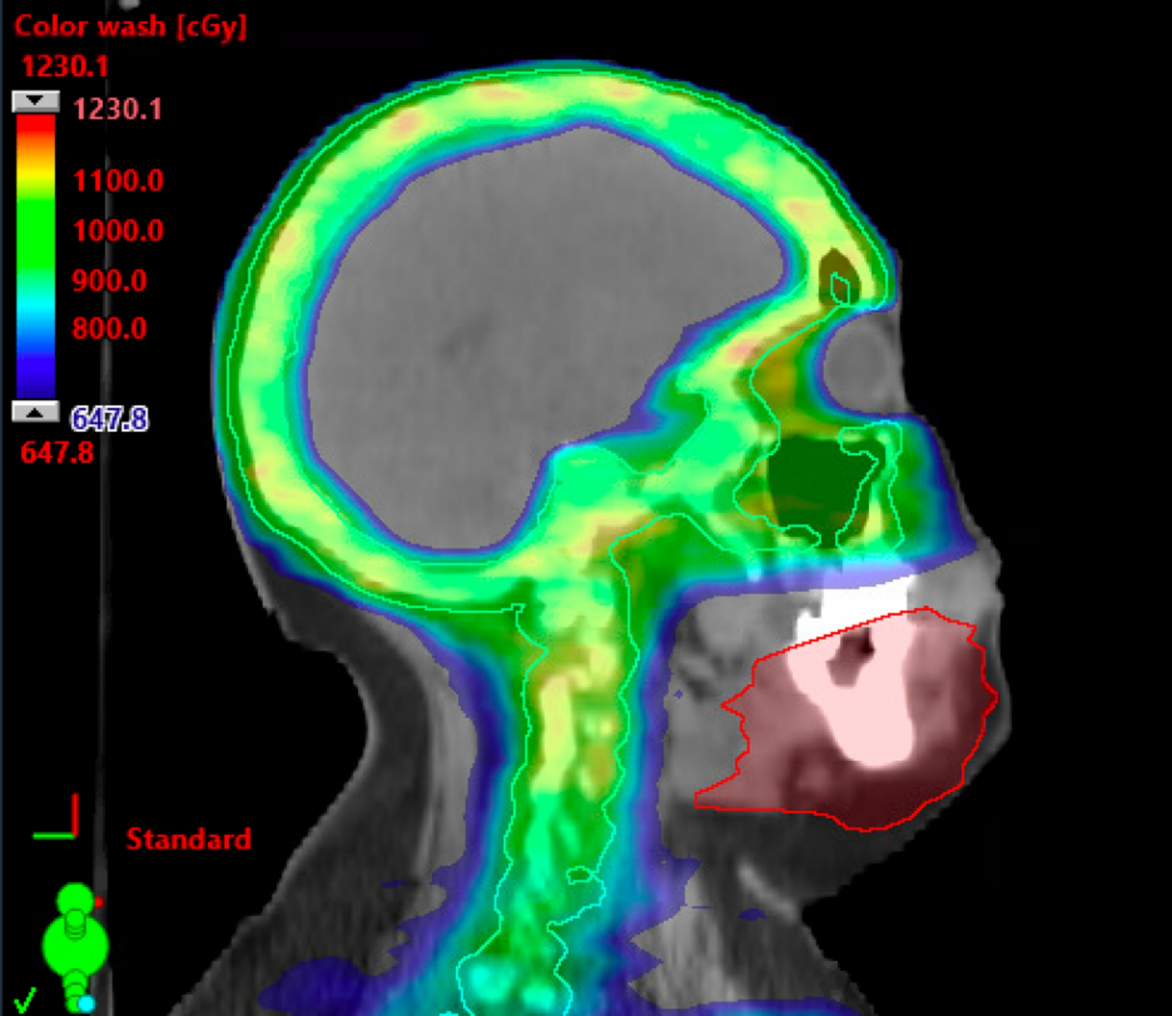
Fusion of her original radiation treatment plan with delineated site of recurrence, which shows the mandibular site of relapse lying outside the prescription dose.

While VMAT provides the opportunity to provide higher doses to a conformal target, it conversely and intentionally leads to lower doses outside of the region of interest. Typical TMI techniques spare the mandible and maxillary bones to minimize the oral cavity dose and risk of mucositis [6]. Current protocols also spare the mid-femur due to technical setup feasibility and the perceived minimal bone marrow involvement of this site. In this case, both of her sites of disease relapse were not included in the initially treated target volume. Figure 4 shows the fusion of her CT scan following treatment with the radiation treatment plan and shows the delineated mandibular site of relapse lying outside the prescription dose. This is the first report of a patient with an out-of-field failure when utilizing a TMI technique and can help inform clinical target volume design for patients undergoing total marrow radiation in the future.

With respect to the management of myeloid sarcoma, there is no standardized approach. Per the International Radiation Oncology Group consensus guidelines, radiation in the setting of a myeloid sarcoma is typically reserved for an inadequate response to chemotherapy and isolated recurrence after HSCT as well as for palliation [16]. Typically, radiation for extramedullary disease in the setting of transplantation is a function of the clinical scenario and the preference of the bone marrow transplant team. At some institutions, radiation is used before transplantation to minimize disease burden, but in other institutions radiation is reserved after transplantation to eradicate any residual disease. At our institution, we reserve radiation for residual disease. In her case, she had an excellent response to chemotherapy and thus no further radiation beyond transplant protocol was felt to be beneficial.

TMI in itself has been explored as a potential risk factor for the development of myeloid sarcoma. The cumulative incidence of extramedullary relapse is about 11.4% at two years [17] when utilizing a TMI technique. It could be argued that the use of TMI may allow extramedullary relapse because of the presence of malignant cells harbored outside of the bone marrow. However, in our previously published study, we found that patients who received TMI received about 50% of this dose delivered to other organs on average [11]. Recently, Kim et al. found a rate of extramedullary relapse that was consistent between TMI and total body irradiation techniques, which clinically supports these dosimetric findings [17]. Of note, their study found that the greatest predictor for extramedullary relapse was pretransplantation extramedullary disease as it was the case in our patient. The mandibular site of relapse may support the rationale for irradiation of extramedullary disease concurrently with TMI. However, the timing of her failure in addition to radiographic evidence of mandibular erosion is suggestive of a bone marrow failure that led to extramedullary involvement. Moreover, extramedullary irradiation alone would not have addressed her right ankle site of relapse. 

Regarding mucositis risk, TMI dosing and myeloablative chemotherapy regimens vary per institutional protocol. Our lower dose 9 Gy in 1.5 Gy twice daily fraction regimen prescribed to include the mandible would dosimetrically result in a similar D10 to the oral cavity as a 15 Gy in 1.5 Gy twice daily fraction regimen prescribed to avoid the mandible, but a higher D50 to the oral mucosa [8,11]. Overall, in spite of the lower total dose, the higher D50 to the oral mucosa would likely lead to higher rates of mucositis if the mandible is included in the TMI target delineation [8].

Overall, the highest rate of failure following TMI transplantation or total body irradiation is within the bone marrow with a cumulative incidence ranging from 22% to 24.8% [18] and accounts for roughly 74% of disease relapse. In the case of TMI, most published series do not report whether bone marrow failure occurs within field or out of the treatment field [5,7,10,11,17,19,20]. In studies where specifics of failure were available, no sites of non-irradiated bone marrow failures were observed [17]. Patients included in these studies are typically at higher risk, which may increase their radioresistant properties and thereby increase the risk of in-field failure. Our patients relapse within the non-irradiated ankle and mandible provide a rationale for inclusion of these sites in the clinical target volume. 

## Conclusions

TMI allows for higher doses to the bone marrow and limits toxicity to other organs at risk such as the brain, kidneys, heart, and lungs. In patients undergoing total marrow radiation, the mandible and distal lower extremities are often not included in the clinical treatment volume due to the low perceived bone marrow involvement of these sites and concerns for morbidity or technical setup feasibility. Herein we report a case of mandibular relapse and lower extremity relapse in a patient who underwent TMI which did not include these two sites. These findings can be helpful to modify treatment planning volumes in the future to include these two areas with the hope to further reduce the out-of-field bone marrow failure rates, while continuing to allow for the dosimetric and clinical advantages of total marrow radiation.
